# Urinary GADD45G Protein Excretion Is Associated with IgA Nephropathy Progression

**DOI:** 10.3390/biomedicines12122846

**Published:** 2024-12-14

**Authors:** Min-Jeong Lee, Hyunee Yim, Ji Eun Park, Inwhee Park, Heungsoo Kim, Gyu-Tae Shin

**Affiliations:** 1 Department of Nephrology, Ajou University School of Medicine, Suwon 16499, Republic of Korea; mjleenephro@aumc.ac.kr (M.-J.L.); 500215@aumc.ac.kr (J.E.P.); inwhee@aumc.ac.kr (I.P.); nephrohs@aumc.ac.kr (H.K.); 2 Department of Pathology, Ajou University School of Medicine, Suwon 16499, Republic of Korea; snoopy@aumc.ac.kr; 3 Department of Endocrinology & Metabolism, Ajou University School of Medicine, Suwon 16499, Republic of Korea

**Keywords:** growth arrest and DNA damage 45G (GADD45G), kidney biomarker, chronic kidney disease, IgA nephropathy

## Abstract

**Background**: Growth arrest and DNA damage 45G (GADD45G) is a family of proteins involved in DNA damage response and cell growth arrest. In this study, we show evidence that urinary GADD45G protein is associated with the progression of IgA nephropathy. **Methods**: Patients diagnosed with IgA nephropathy without reversible acute kidney injury at study initiation and with at least one subsequent serum creatinine (SCr) measurement were included. A 50% or greater increase in SCr level was used as an endpoint for the deterioration of renal function. Enzyme-linked immunosorbent assay (ELISA) was performed using a Human GADD45G ELISA kit. Renal biopsy tissues were stained with a monoclonal mouse anti-GADD45G antibody. **Results**: Forty-five patients whose renal biopsy revealed IgA nephropathy were enrolled. Urinary GADD45G and urinary protein concentrations were 1.26 [0.69–2.20] μg/g creatinine and 0.65 [0.24–1.60] g/g creatinine, respectively. Urinary GADD45G showed significant positive correlations with SCr-slopes and urinary protein. The SCr-slope of the highest tertile group of urinary GADD45G (above 1.95 μg/g creatinine) was significantly higher than that of the lowest tertile group (below 0.90 μg/g). Univariate Cox regression analysis showed that urinary GADD45G was significantly associated with deterioration of renal function. A Kaplan–Meier test showed a significant difference in event-free survival for deterioration of renal function between the highest urinary GADD45G tertile group and other tertile groups. The area under the receiver operating characteristics (ROC) curve indicated urinary GADD45G had a good performance in predicting renal outcome (cut-off point 1.67 μg/g, positive predictive value 36.8%, negative predictive value 100%). Immunohistochemistry showed that GADD45G was expressed across all pathologic grades of IgA nephropathy and mainly detected in the cytoplasm of renal tubules, whereas no staining was noted in normal tissues. **Conclusions**: Urinary GADD45G excretion was significantly associated with kidney disease progression in patients with IgA nephropathy.

## 1. Introduction

Growth arrest and DNA damage 45G (GADD45G) is a stress-responsive molecule that plays a role in the activation of checkpoints of the cell cycle [1] and the induction of apoptosis [2].

IgA nephropathy is the most common type of primary glomerulonephritis worldwide, accounting for more than 40% of primary glomerulonephritis in Asia [3]. Progression to end-stage renal disease (ESRD) develops in a substantial proportion of patients with IgA nephropathy over the long term. A recent multicenter study in Korea has shown that progression to ESRD occurs in approximately 7.2% at a median follow-up of 6.4 years after renal biopsy [4]. Previous studies have shown that renal outcome appears to correlate more with tubulointerstitial fibrosis than with glomerulosclerosis, although the pathogenesis of IgA nephropathy starts with mesangial deposition of IgA [5,6]. Evidence has shown that apoptosis might be the key process in the development of tubulointerstitial fibrosis [7], where tubular injury initiates the apoptotic cascade, which leads to tubular atrophy and deposition of extracellular matrix, resulting in tubulointerstitial fibrosis [8]. Accordingly, it has been shown that apoptosis of renal tubular cells per se is associated with the progression of IgA nephropathy [9].

We have previously demonstrated that GADD45G is implicated in renal tubular injury in a rodent model [10] and that GADD45G plays a critical role in apoptotic pathways of renal tubular cells [11]. With regard to IgA nephropathy, we have demonstrated that GADD45G mRNA expression in urinary sediment cells is associated with a decline in renal function [12]. In the present study, we extend such findings by demonstrating that urinary GADD45G protein excretion was associated with kidney disease progression in patients with IgA nephropathy.

## 2. Materials and Methods

### 2.1. Study Population

This study was conducted with a cohort of patients who underwent kidney biopsy at a single-center tertiary university hospital. Urine samples were collected from patients aged 18 years and more who gave consent on the day of admission for percutaneous renal biopsy. Samples were transported to the institutional biobank for storage in ultra-low temperature archives for potential future analysis. For this study, urine samples of patients who met the following inclusion criteria were retrieved: patients with IgA nephropathy if they did not have reversible acute kidney injury (AKI) at the time of renal biopsy with having at least one follow-up serum creatinine (SCr) measurement. Patients were categorized to have AKI if they had initial SCr values above normal, which dropped 50 percent or more during the follow-up period. Urine samples were divided into two aliquots for measuring protein and creatinine at the Department of Clinical Laboratory and for measuring GADD45G at the authors’ laboratory. Urinary GADD45G and protein concentrations were expressed as μg/g creatinine and g/g creatinine, respectively, as they were normalized by urine creatinine concentrations. Data for urinary creatinine and protein, as well as serum albumin and cholesterol, were retrieved from routine clinical laboratory reports available in patients’ medical records. These parameters were not measured separately for this study but were used as reported from the hospital laboratory. Paraffin-embedded blocks were retrieved from the archives of the Department of Pathology. Clinical data were obtained from patients’ medical records starting from the date of urine sample collection (study entry). In addition, the MEST (M: mesangial hypercellularity; E: endocapillary hypercellularity; S: segmental glomerulosclerosis; T: tubular atrophy/interstitial fibrosis) score of the Oxford classification [13] was obtained from patients’ biopsy reports. This study was approved by the Institutional Review Board of Ajou University Medical Center (AJOUIRB-KSP-2020-237) and was performed in accordance with current guidelines and regulations of the Helsinki Declaration. Written informed consent was obtained from enrolled patients.

### 2.2. Enzyme-Linked Immunosorbent Assays (ELISA)

To measure the GADD45G protein in urine, ELISA was performed using a Human GADD45G ELISA kit (MBS 2884626, MyBioSource, San Diego, CA, USA) according to the manufacturer’s protocols. The detection range of the kit is 78–5000 pg/mL, with a sensitivity of less than 39.2 pg/mL. Performance characteristics include an intra-assay coefficient of variation (CV) of ≤6.0%, an inter-assay CV of ≤7.9%, and a spike average recovery of 84%. Briefly, urine was centrifuged at 1000× *g* for 20 min at 4 °C to remove urinary sediment, and the supernatant was used for analysis. Urine samples (100 μL) were loaded into microplate wells and incubated at 37 °C for 2 h followed by the addition of 100 μL biotin-conjugated antibody preparation for an additional 1 h at 37 °C. Avidin conjugated to horseradish peroxidase (HRP) was then added to each microplate well and incubated at 37 °C for 1 h. The reaction was visualized by adding 90 μL chromogenic substrate (TMB), followed by incubation at 37 °C for 15 min. The color development was stopped by adding 50 μL sulfuric acid solution. Color change was measured using a microplate reader at a wavelength of 450 nm.

### 2.3. Immunohistochemistry

Renal biopsy tissues were first deparaffinized and subjected to heat-induced antigen retrieval by incubation in 0.01 M citrate (pH 6.0) buffer at 95 °C for 30 min. Endogenous peroxidase was blocked with a peroxidase-blocking solution (Agilent Technologies, Santa Clara, CA, USA) for 10 min. The tissue was then incubated with a monoclonal mouse anti-GADD45G antibody (SC-393261, Santa Cruz Biotechnology, Santa Cruz, CA, USA) diluted at a concentration of 1:100 at room temperature for one hour in a humidified chamber. The tissue section was then incubated with a biotinylated horse antimouse secondary antibody for 30 min, followed by incubation with complexes formed by avidin and biotinylated horseradish peroxidase (HRP) for 30 min (Vectastain Elite ABC kit) (Vector Laboratories, Burlingame, CA, USA). Peroxidase activity was visualized using 3,3′-diaminobenzidine (DAB) for two minutes, and then the tissue was counterstained with hematoxylin. Control specimens were taken from normal parts of kidneys from patients who underwent surgeries for renal tumors. The slide scan images were obtained using the slidescanner (Axio Scan Z1, Carl Zeiss, Göttingen, Germany) and the ZEN 3.1 imaging software (Carl Zeiss). DAB-stained area was quantified using Fiji version 1.53k of ImageJ (http://fiji.sc accessed on 29 October 2021). The optical density (OD) was calculated as follows: OD = log (255/mean intensity), where mean intensity for negative staining was 255.

### 2.4. Statistical Analysis

Data were expressed as median and interquartile range (IQR) for non-normally distributed data and as mean ± standard deviation (SD) for normally distributed data. Student’s *t*-test or one-way analysis of variance (ANOVA) followed by Bonferroni’s post hoc comparison test was used to compare continuous variables. Non-parametric tests such as the Kruskal–Wallis test were used for non-normally distributed variables, and pairwise comparisons were conducted using the Mann–Whitney U test. Follow-up time for each patient was calculated from the biopsy date until the date of the primary endpoint or until the final follow-up. A 50% or greater increase in SCr level was used as the primary endpoint of deterioration of renal function. Linear regression analysis by the least-squares method was used to determine the slopes of SCr over time for each patient. The degree of association between variables was determined using the Pearson correlation coefficient (r). A univariate Cox proportional hazards survival model was used to estimate hazard ratios with 95% confidence intervals. Survival curves were estimated for each group using the Kaplan–Meier analysis, where pairwise comparisons were made using the log-rank test. Receiver operating characteristic curve (ROC) analysis was performed to determine the variables’ predictive values for renal survival. A *p*-value < 0.05 was considered significant. All statistical analyses were performed using IBM SPSS Statistics version 24 (IBM, Armonk, NY, USA).

## 3. Results

### 3.1. Patients’ Characteristics

A total of 45 patients were selected for this study. All included patients were those who had been referred to our renal clinic for further work-up by their primary care physicians. Of these enrolled patients, 10 (22.2%) received immunosuppressive therapy, including corticosteroids and/or cyclosporine A sometime during the course of follow-up. The mean follow-up was 593.6 ± 253.6 days (range 15–1065 days). Urinary GADD45G and protein concentrations were divided by urine creatinine concentrations to eliminate the effect of the concentration status of urine. Urinary GADD45G was detectable in 44 of 45 patients. Median values of urinary GADD45G and protein were 1.26 [0.69–2.20] μg/g creatinine and 0.65 [0.24–1.60] g/g creatinine, respectively, indicating that the amount of GADD45G was one-millionth the amount of total protein. Baseline characteristics of patients are listed in Table 1.

### 3.2. Correlation Between Urinary GADD45G Concentrations and Clinical Parameters

The correlation matrix among urinary GADD45G, SCr-slopes, urinary protein, age, initial SCr, body mass index (BMI), serum albumin, and serum cholesterol is shown in Table 2. Urinary GADD45G showed a positive correlation with SCr-slopes (r = 0.372, *p* = 0.012) and a strong positive correlation with urinary protein (r = 0.774, *p* < 0.001). In addition, urinary GADD45G correlated positively with age (r = 0.340, *p* = 0.022) but negatively with serum albumin (r = −0.651, *p* < 0.001). Similar results were obtained for a subset of patients who had never received immunosuppressive therapy (N = 35) as follows. Urinary GADD45G showed positive correlations with SCr-slopes (r = 0.487, *p* = 0.003) and urinary protein (r = 0.738, *p* <0.001). In addition, urinary GADD45G correlated positively with age (r = 0.336, *p* = 0.048) but negatively with serum albumin (r = −0.620, *p* < 0.001).

**Figure 1:**
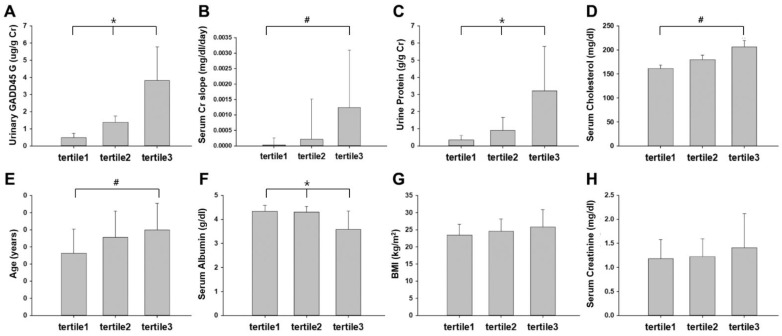
Comparison of clinical parameters between urinary GADD45G tertiles: Tertile3 showed significant differences in urinary GADD45G (**A**), urinary protein (**C**), and serum albumin (**F**) in comparison with both Tertile1 and Tertile2. Tertile3 also showed significant differences in SCr slope (**B**), serum cholesterol (**D**), and age (**E**) in comparison with tertile1. There was no significant difference in BMI (**G**) and initial SCr (**H**) between groups. * *p* < 0.05 versus Tertile1 and Tertile2; # *p* < 0.05 versus tertile1. Total patients, N = 45. Tertiles of urinary GADD45G (N = 15 for each tertile): Tertile1, lowest; Tertile2, middle; Tertile3, highest. SCr, serum creatinine; BMI, body mass index.

To further characterize the relationship of urinary GADD45G with clinical parameters, we divided patients into three subgroups according to their respective tertiles of urinary GADD45G concentrations (tertile1, urinary GADD45G < 0.90 μg/g creatinine; Tertile2, 0.90 ≤ urinary GADD45G < 1.95 μg/g creatinine; Tertile3, urinary GADD45G ≥ 1.95 μg/g creatinine). The highest tertile group showed significantly higher urinary GADD45G levels than the lowest tertile group (*p* < 0.001) and the middle (*p* < 0.001) tertile group (Figure 1A). The SCr-slope of the highest tertile group was significantly higher than that of the lowest tertile group (*p* = 0.046), indicating that renal function deteriorated more rapidly in the highest tertile group (Figure 1B). Urinary protein was significantly higher in the highest tertile group than in the middle tertile (*p* = 0.009) and the lowest tertile (*p* < 0.001) groups. Additionally, significant differences were observed between the middle and lowest tertile groups (*p* = 0.005) (Figure 1C). In addition, serum cholesterol levels and patients’ age were significantly higher in the highest tertile group than in the lowest tertile group (*p* = 0.025 and *p* = 0.049, respectively) (Figure 1D,E). In contrast, serum albumin levels were significantly lower in the highest tertile group than in the middle (*p* = 0.001) and lowest tertile (*p* < 0.001) groups (Figure 1F). BMI and initial SCr were comparable between groups (Figure 1G,H). In a subset of patients who had never received immunosuppressive therapy (N = 35, No-ISA group), the difference in the SCr-slope of each tertile did not reach statistical significance due to reduced number of patients (*p* = 0.061).

### 3.3. Association of Urinary GADD45G Concentration with Renal Prognosis

As shown in Table 3, univariate Cox regression analysis showed that urinary GADD45G was significantly associated with deterioration of renal function (HR, 1.63; *p* = 0.010). In accordance with previous studies on IgA nephropathy, proteinuria (HR, 1.89; *p* < 0.001) [14], age (HR, 1.060; *p* = 0.023) [15], serum albumin (HR, 0.31; *p* = 0.002) [16] and serum cholesterol (HR, 1.02; *p* = 0.007) [17] were also significantly associated with deterioration of renal function. On the other hand, in the No-ISA group, urinary GADD45G was the only variable that was associated with deterioration of renal function in the same analysis (HR, 1.14; *p* = 0.025).

**Figure 2:**
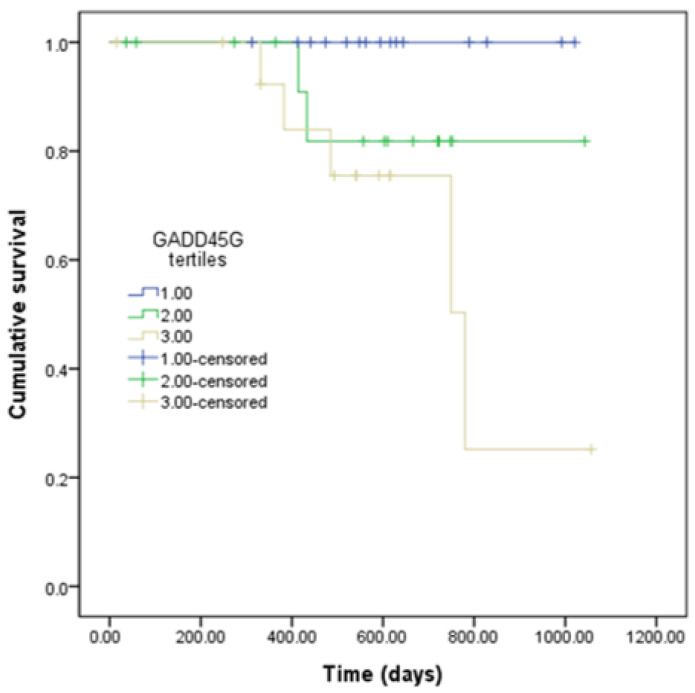
Kaplan–Meier estimates of renal survival based on tertiles of urinary GADD45G levels. The endpoint was an increase of 50% or more in SCr concentrations above baseline. Comparisons between groups were made using the log-rank test with pairwise post hoc comparisons. Total patients, N = 45. Tertiles of urinary GADD45G (N = 15 for each tertile): Tertile1, lowest; Tertile2, middle; Tertile3, highest. SCr, serum creatinine.

Kaplan–Meier test showed a significantly worse renal survival in the highest urinary GADD45G tertile group than in the lowest tertile group (*p* = 0.010) (Figure 2). However, renal survival was comparable between the highest urinary GADD45G tertile group and the middle tertile group (*p* = 0.355) (Figure 2). Similar results were obtained in the no-ISA group: highest versus lowest (*p* = 0.047) and highest versus middle (*p* = 0.082).

**Figure 3:**
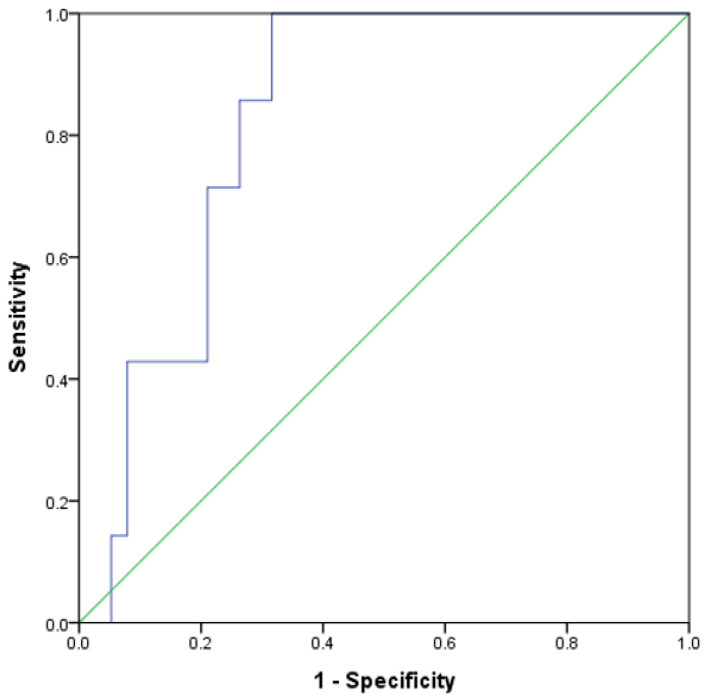
The area under the receiver operating characteristics (ROC) curve of urinary GADD45G. The endpoint is an increase in SCr concentration of at least 50% above baseline. The blue and the green lines represent the ROC curve and the random classifier, respectively. The points above the green line indicate a better probability than the random one. Total patients, N = 45.

The area under the ROC curve of urinary GADD45G was 0.827 (*p* = 0.006), indicating that GADD45G had a good performance in predicting renal outcome (Figure 3). The cutoff value of 1.67 μg/g was determined from the ROC curve at which the Youden index (sensitivity + specificity–1) was the maximum [18]. The positive predictive value (PPV) of this cutoff was 36.8%, and the negative predictive value (NPV) was 100%. In the no-ISA group, the area under the ROC curve of urinary GADD45G was 0.938 (*p* = 0.013). At a cutoff value of 1.98 μg/g for urinary GADD45G, the PPV was 37.5%, and the NPV was 100%.

### 3.4. Renal Pathologic Features and Immunohistochemical Staining of GADD45G

**Figure 4:**
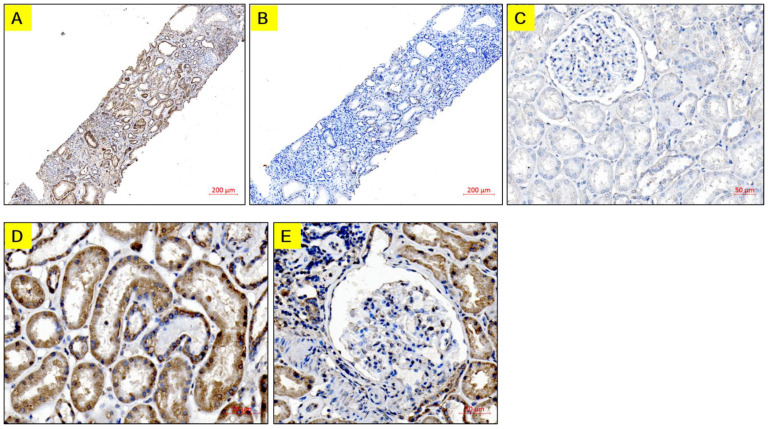
Immunohistochemistry expression of GADD45G: Biopsy sections from the same patient with IgA nephropathy were incubated with a primary antibody (**A**) or without a primary antibody (**B**), with the negative control (**B**) showing the lack of DAB staining. (**C**) There was little or no staining in the normal tissue taken from normal parts of removed kidneys for renal tumors. (**D**,**E**) Representative examples of GADD45G staining for specimens of IgA nephropathy. (**D**) There was an extensive expression of GADD45G in the cytoplasm of tubular cells. (**E**) Mild GADD45G staining was also noted in Bowman’s epithelial cells and some glomerular cells. Tissues were stained with DAB (brown) and counterstained with hematoxylin (blue). The scale bar was produced by the ZEN imaging software according to the magnification.

**Figure 5:**
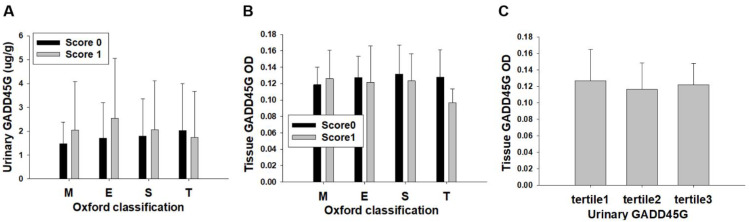
Relationships of pathologic grades with urinary and tissue GADD45G: (**A**) Urinary GADD45G levels according to the Oxford classification. (**B**) Measured OD of GADD45G staining according to the Oxford classification. (**C**) OD of GADD45G staining according to their respective tertiles of urinary GADD45G concentrations. Tertiles of urinary GADD45G (N = 15 for each tertile): Tertile1, lowest; Tertile2, middle; and Tertile3, highest.

Immunohistochemistry showed extensive GADD45G staining in biopsies of all 45 IgA nephropathy patients (Figure 4A). To determine the degree of false positive staining, sections were incubated without a primary antibody where only non-specific faint signals were detected (Figure 4B). To compare the GADD45G expression of IgA patients with normal kidney tissue, the analysis was performed using normal tissues from the nephrectomy specimen where only non-specific background staining was noted (Figure 4C). GADD45G staining was mainly identified in the cytoplasm of renal tubules (Figure 4D) and in some cells of glomeruli (Figure 4E). The Oxford classification was available for 36 patients: M (M0/M1), 4/32 (11.1%/88.9%); E (E0/E1), 23/13 (63.8%/36.2%); S (S0/S1), 8/28 (28.5%/71.5%); T (T0/T1), 33/3 (91.6%/8.4%). Comparisons of urinary and tissue GADD45G were made between M0 versus M1, E0 versus E1, S0 versus S1, and T0 versus T1 of the Oxford classification [19,20]. Results show that urinary GADD45G levels and measured OD of GADD45G staining were not different between these pathologic scores (Figure 5A,B). Contrary to our expectation, the OD of GADD45G staining neither correlated with urinary GADD45G (r = 0.023, *p* = 0.882) nor differed among urinary GADD45G tertile groups (Figure 5C).

## 4. Discussion

Our data indicate that elevated urinary GADD45G levels are associated with poor renal outcomes in patients with IgA nephropathy. We have previously shown that GADD45G mRNA expression in urinary sediment is associated with kidney disease progression in patients with IgA nephropathy [12]. The present study was different from the previous study in that the measurement of GADD45G protein in urine by ELISA in the present study enabled us to quantify relationships between urinary GADD45G levels and various clinical characteristics. In the present study, we chose the SCr slope as a primary endpoint of deterioration of renal function rather than eGFR, considering that we monitor changes in SCr as the surrogate of renal function changes in our practice, which is the most intuitive way of assessing changes in patients’ renal function. In fact, there are many studies that have used the SCr slope to track the progression of renal function. We found that elevated urinary GADD45G concentrations were significantly correlated with worsening renal function, as measured by the SCr slope [21,22]. There were no previous studies that measured GADD45G protein in urine samples. We expect that more studies will follow our study to see the implications of the urinary GADD45G protein in renal diseases.

We have previously shown that GADD45G contributes to renal tubular cell apoptosis [11]. In the present study, GADD45G was identified in the cytoplasm of renal tubular cells of IgA nephropathy across all histological grades. Other investigators have shown that apoptosis of renal tubular cells is associated with a decline of renal function in IgA nephropathy [9]. Taken together, it can be hypothesized that GADD45G is induced in renal tubular cells from the early pathogenic process of IgA nephropathy, which subsequently contributes to the occurrence of apoptosis of renal tubular cells, thereby leading to the worsening of renal function.

GADD45G is not a secretory protein. Apoptotic cells do not release cellular contents since clearance of apoptotic cells by phagocytes occurs without disruption of the plasma membrane [23]. If that is the case, one may wonder how GADD45G can appear in urine. The following observations may provide an answer to this question. First, microparticles released from apoptotic cells lose their cell membrane integrity rather quickly, resulting in a leak of intracellular contents into the microenvironment [24]. In addition, secondary necrosis can be developed in tissues where the clearance capacity of phagocytes is overwhelmed or reduced [25]. It is well known that plasma membrane rupture by necrotic process can lead to the release of cellular contents into the microenvironment [26].

Another finding to note in the present study was that urinary GADD45G concentrations correlated with urinary protein concentrations. The amount of urinary GADD45G excretion only accounted for a millionth of the amount of urinary protein excretion. Therefore, such a correlation should not be simply because urinary GADD45G adds to the amount of urinary protein. The initiating event in the pathogenesis of IgA nephropathy occurs in the glomeruli. Tubulointerstitial damage then follows. Albumin filtered through diseased glomeruli accounts for the majority (60–70%) of urinary protein composition in IgA nephropathy. Urinary albumin is a well-known predictor of progression of IgA nephropathy [27]. Given that albumin per se in tubular flow can induce tubular apoptosis [28], filtered albumin might induce GADD45G in tubular cells, thereby showing a close relationship between urinary protein and GADD45G in our study. Urinary GADD45G might also be a marker of more severe disease like proteinuria [29], which can result in a significant association between these two proteins without a direct interaction.

It was noteworthy that GADD45G expression was identified across all histological grades of biopsies in contrast to the negative staining in normal kidney samples. Accordingly, almost all urine samples showed detectable levels of GADD45G. These findings suggest that DNA damaging stress to tubular cells might occur even before abnormal histologic findings of tubular cells are detectable by light microscopy in IgA nephropathy. In fact, it has been postulated that there is a glomerulotubular cross-talk that operates upon mesangial IgA deposition in an early stage of IgA nephropathy, culminating in tubular cell death and fibrosis [30].

With regard to the relationship between urinary GADD45G level and its quantitative tissue expression, we were unable to find correlations between these variables. This might be due to the fact that each biopsy is composed of different areas of kidneys with relatively small tissue sizes, whereas urinary GADD45G levels reflect the whole kidney area. Accordingly, other investigators have also reported no association between urinary biomarker values and quantitative tissue stainings [31].

One limitation of our study is the limited applicability of our results to clinical practice arising from the complexity of measuring urinary GADD45G concentrations using ELISA compared with standard urine protein measurements. Urine protein concentration is a well-established and reliable indicator of the progression of IgA nephropathy, and a significant correlation has been observed between urine protein levels and renal function decline. Therefore, although GADD45G provides detailed insight into the pathogenesis of renal function decline in IgA nephropathy, it remains realistically challenging to replace the cost-effective and rapid measurement of urine protein with urinary GADD45G concentration as a practical indicator in clinical settings. Another limitation is that tissue biopsy results were not directly correlated with urinary GADD45G levels, so further studies are needed to clarify how urinary GADD45G reflects specific kidney regions. Unfortunately, in clinical practice in Korea, the spot urine protein-to-creatinine ratio is commonly used instead of measuring albuminuria. This is because the Health Insurance Review and Assessment (HIRA) restricts test prescriptions to cases other than microalbuminuria, and measuring albuminuria in patients with high proteinuria is considered unnecessary, which can lead to reduced reimbursements. Unfortunately, this meant that serial follow-up of albuminuria was not conducted in our study, and we were unable to include these data in our population. There is also a lack of information on the racial or ethnic differences in GADD45G expression. Although GADD45G has shown potential as a biomarker in IgA nephropathy, there is currently limited research on whether its expression differs significantly by race or ethnicity. As IgA nephropathy is more prevalent in certain populations, such as East Asian populations, further studies are needed to investigate whether GADD45G expression varies across different racial or ethnic groups. This will help determine whether GADD45G can be a universally applicable biomarker or whether its clinical utility may be more specific to certain populations.

In conclusion, we showed that GADD45G protein excretion in urine was associated with worsening renal function in IgA nephropathy. We also found that GADD45G was expressed in renal tubules across all pathologic grades, indicating that tubular damage was an early pathogenic process of IgA nephropathy. Further studies are needed to establish the relationship between urinary GADD45G level and the progression of IgA nephropathy and define the role of GADD45G in the pathogenesis of IgA nephropathy.

## Figures and Tables

**Table 1:** Baseline characteristics of patients.

Characteristics	N = 45	Range
Male (%)	26 (57.8)	–
Urinary GADD45G, μg/g creatinine	1.26 [0.69–2.20]	0.00–9.03
Urinary protein, g/g creatinine	0.65 [0.24–1.60]	0.06–8.18
Serum albumin, g/dL	4.07 ± 0.58	1.70–4.90
Age, years	43.97 ± 15.65	18–78
Serum cholesterol, mg/dL	182.57 ± 47.60	95–326
SCr, mg/dL	1.26 ± 0.51	0.52–2.64
Body mass index, kg/m^2^	24.61 ± 4.10	18.35–39.90

Continuous variables are presented as mean ± SD, and urinary protein and urinary GADD45G are presented as median [25th quartile–75th quartile]. Categorical variables are presented as N (%). SCr, serum creatinine.

**Table 2:** Correlations of urinary GADD45G with clinical parameters.

	Urinary GADD45G	SCr-Slope	Urinary Protein	Age	BMI	SCr	Albumin	Cholesterol
Urinary GADD45G	1	0.372 *	0.774 **	0.340 *	0.28	0.256	−0.651 **	0.066
SCr-slope	0.372 *	1	0.375 *	0.243	0.08	0.406 **	−0.329 *	0.171
Urinary protein	0.774 **	0.375 *	1	0.285	0.28	0.126	−0.849 **	0.320 *
Albumin	−0.651 **	−0.329 *	−0.849 **	−0.408 **	−0.20	−0.158	1	−0.321 *
Age	0.340 *	0.243	0.285	1	−0.02	0.346 *	−0.408 **	0.011
Cholesterol	0.066	0.171	0.320 *	0.011	0.06	−0.262	−0.312 *	1
SCr	0.256	0.406 **	0.126	0.346 *	0.17	1	−0.158	−0.262
BMI	0.276	0.08	0.284	−0.023	1	0.167	−0.199	0.058

Data are presented as correlation coefficients of Pearson analysis (N = 45). * *p* < 0.05 (bivariate); ** *p* < 0.01 (bivariate). SCr, serum creatinine; BMI, body mass index.

**Table 3:** Univariate Cox proportional hazard regression analysis for renal prognosis.

Variables	HR (95% CI)	*p* Value
Urinary GADD45G	1.63 (1.12–2.36)	0.010
Urine protein	1.89 (1.34–2.66)	<0.001
Age	1.06 (1.01–1.12)	0.023
Serum Albumin	0.31 (0.14–0.66)	0.002
Serum cholesterol	1.02 (1.01–1.03)	0.007
BMI	1.04 (0.90–1.19)	0.628
SCr	2.16 (0.61–7.58)	0.230
Male	3.25 (0.67–15.84)	0.145

A 50% or greater increase in SCr level was used as the endpoint of deterioration of renal function. Total patients, N = 45. HR, hazard ratio; CI, confidence interval; SCr, serum creatinine at baseline; BMI, body mass index.

## Data Availability

The original contributions presented in this study are included in the article. Further inquiries can be directed to the corresponding author.

## Abbreviations

GADD45G = Growth arrest DNA damage 45G, SCr = serum creatinine, ELISA = Enzyme-linked immunosorbent assay, ESRD = end-stage renal disease, AKI = acute kidney injury, HRP = horseradish peroxidase, TMB = chromogenic substrate, DAB = diaminobenzidine, SD = standard deviation, ANOVA = one-way analysis of variance, ROC = Receiver operating characteristic curve, PPV = positive predictive value, NPV = negative predictive value.
